# Prognostic implications of various models for calculation of S-phase fraction in 259 patients with soft tissue sarcoma

**DOI:** 10.1038/sj.bjc.6690193

**Published:** 1999-03

**Authors:** P Gustafson, B Baldetorp, M Fernö, M Åkerman

**Affiliations:** Department of Orthopedics, University Hospital, Lund, SE-221 85, Sweden; Department of Oncology, University Hospital, Lund, SE-221 85, Sweden; Department of Pathology and Cytology, University Hospital, Lund, SE-221 85, Sweden

**Keywords:** soft tissue sarcoma, DNA flow cytometry, S-phase fraction, background correction, prognosis

## Abstract

The S-phase fraction (SPF) in flow cytometric DNA histograms in soft tissue sarcoma (STS) can be calculated in various ways. The traditional planimetric method of Baisch has been shown to be prognostic, but is hampered by a failure rate of around 40%. We therefore tested other models to see if this rate could be decreased with retained prognostic value. In 259 STS of the locomotor system the SPF was calculated according to Baisch and with commercial parametric MultiCycle software using different corrections for background. Using the Baisch model, 159 histograms could be evaluated for SPF. The 5-year metastasis-free survival rate (MFSR) was 0.94 for the low-risk group (defined with SPF), and 0.53 for the high-risk group. In the low-risk group, four of the seven patients who developed metastasis did so after 5 years. Using the MultiCycle software, SPF could be calculated in 253 tumours. Depending on type of background correction used, the 5-year MFSR varied between 0.67 and 0.82 for the low-risk group, and between 0.47 and 0.53 for the high-risk group. The late metastasis pattern in the low-risk group was never seen using the MultiCycle software. We conclude that in paraffin archival material, calculation of SPF according to Baisch is preferable in clinical use due to better separation between low-risk and high-risk groups, and also the possibility to identify patients who metastasize late. © 1999 Cancer Research Campaign


*British Journal of Cancer* (1999) **79**(7/8), 1205–1209
© 1999 Cancer Research Campaign
Article no. bjoc.1998.0193

### Prognostic implications of various models for calculation of S-phase fraction in 259 patients with soft tissue sarcoma

**P Gustafson[1], B Baldetorp[2], M Fernö[2] and M Åkerman[3]**

Departments of [1]Orthopedics, [2]Oncology and [3]Pathology and Cytology, University Hospital, SE-221 85 Lund, Sweden

**Summary** The S-phase fraction (SPF) in flow cytometric DNA histograms in soft tissue sarcoma (STS) can be calculated in various ways. The traditional planimetric method of Baisch has been shown to be prognostic, but is hampered by a failure rate of around 40%. We therefore tested other models to see if this rate could be decreased with retained prognostic value. In 259 STS of the locomotor system the SPF was calculated according to Baisch and with commercial parametric MultiCycle software using different corrections for background. Using the Baisch model, 159 histograms could be evaluated for SPF. The 5-year metastasis-free survival rate (MFSR) was 0.94 for the low-risk group (defined with SPF), and 0.53 for the high-risk group. In the low-risk group, four of the seven patients who developed metastasis did so after 5 years. Using the MultiCycle software, SPF could be calculated in 253 tumours. Depending on type of background correction used, the 5-year MFSR varied between 0.67 and 0.82 for the low-risk group, and between 0.47 and 0.53 for the high-risk group. The late metastasis pattern in the low-risk group was never seen using the MultiCycle software. We conclude that in paraffin archival material, calculation of SPF according to Baisch is preferable in clinical use due to better separation between low-risk and high-risk groups, and also the possibility to identify patients who metastasize late.

**Keywords:** soft tissue sarcoma; DNA flow cytometry; S-phase fraction; background correction; prognosis

Flow cytometric S-phase fraction (SPF) has been shown to have independent prognostic value in soft tissue sarcoma (STS) (Huuhtanen et al, 1996; Collin et al, 1997; Gustafson et al, 1997). However, in these series, SPFs have been calculated in different ways, using different corrections for background in the DNA histograms. The traditional way is the planimetric method of Baisch et al (1975), where the S-phase compartment is assumed to constitute a rectangular distribution between the modal values of the G0/G1 and G2 peaks. This method is hampered by the fact that SPF can only be calculated in six out of ten STS (Collin et al, 1997; Gustafson et al, 1997), the failures primarily due to (1) a high background noise distribution; (2) a small non-diploid peak; and (3) a high coefficient of variation. If one of these criteria is fulfilled, SPF should, according to international consensus guidelines, not be calculated (Shankey et al, 1993). Recently, several computerized programs have been introduced, where the SPF value is calculated using mathematical descriptions of the histogram and the least square curvefitting technique. These models have a theoretical advantage in promising a higher rate in calculation of SPF in histograms with high background and/or small G0/G1 peaks. There is no consensus on which technique for S-phase fraction gives the most accurate and clinically useful SPF value. We therefore examined 259 STS, using the Baisch method and the MultiCycle method, and compared the various models using metastasis-free survival as end point.

*Received 4 March 1998*
*Revised 12 June 1998*
*Accepted 6 August 1998*

*Correspondence to:* P Gustafson

### MATERIAL AND METHODS

### Patients

The population-based database at the Musculoskeletal Tumor Center in Lund, Sweden holds 508 patients with STS of extremity and trunk wall diagnosed between 1964 and 1989. Patients have been identified via the Regional Tumor Registry. The database therefore comprises all patients in the Southern Swedish Health Care Region (1.5 million inhabitants), irrespective of whether the patients have been treated at our institution or at local hospitals in the region. Criteria for inclusion as well as classification of treatment, histopathology, including microscopic tumour necrosis, vascular invasion and malignancy grading, have been described elsewhere (Gustafson, 1994). In 260 of these 508 patients, paraffin-embedded material could be retrieved for flow cytometric DNA analysis. After the DNA analysis was done, one tumour was reclassified from low-grade STS to benign, and thus 259 patients remained for analysis.

The 259 patients had a median age of 64 (range 18–90) years, and a median tumour size of 7 (range 1–40) cm. Two patients had metastasis at diagnosis (one pulmonary, one lymph nodes). Malignant fibrous histiocytoma (MFH) was the commonest histotype, and grade IV (four-grade scale) the commonest malignancy grade (Table 1). All but one patient were operated on; 75 patients had inadequate local treatment (surgery with an intralesional margin with or without radiotherapy or surgery with a marginal margin without radiotherapy: 26 at the Musculoskeletal Tumor Center and 49 at non-Center hospitals), and 183 patients had adequate local treatment (surgery with a marginal margin with radiotherapy or surgery with a wide or radical margin with or without radiotherapy: 158 at the Center and 25 at non-Center hospitals). Twenty-nine patients received chemotherapy, three of whom received it preoperatively. These patients were not separately analysed, since they did not differ from those who did not receive chemotherapy as regards clinico-pathologic factors or outcome.

The median follow-up time for the 89 patients alive at last follow-up was 16 (range 7–32) years. Eighty-three patients developed local recurrence, 42 of the 74 treated outside the Center and 41 of the 184 treated at the Center. At latest follow-up 100 patients had developed distant metastasis, giving a 5-year metastasis-free survival rate (MFSR) of 0.63.

Compared to the population-based database, the 259 patients were a representative subset as regards age, sex, tumour localization, tumour depth, tumour size, malignancy grade, microscopic tumour necrosis, vascular invasion, local treatment, metastasis rate and length of follow-up. In the present series liposarcoma was more common (15% vs 10%) than in the database, and leiomyosarcoma was less common (18% vs 25%).

### Flow cytometric DNA analysis

One representative paraffin-embedded block was chosen for disintegration and analysis. A 4-μm section adjacent to the 100-μm section for disintegration was stained with haematoxylin and eosin and served as control to ensure that preserved and non-necrotic sarcoma tissue was analysed. The DNA analysis was performed principally according to Schutte et al (1985), including treatment with trypsin and staining with propidium iodide. The DNA content in individual nuclei was analysed in an Ortho 50 H cytofluorograph (Baldetorp et al, 1989).

#### *S-phase fraction*

***Baisch*** SPF was calculated with a planimetric method (Baisch et al, 1975) assuming the S-phase compartment to constitute a rectangular distribution between the modal values of the G0/G1 and G2 peaks, and was expressed as the percentage of nuclei in the S-phase of the total number of nuclei. In case of bimodality in the 2C region, i.e. neardiploidy with a DNA index (DI) for the non-diploid stemline below approximately 1.3, a mean SPF value for the diploid and non-diploid stemline was calculated. When the DI exceeded 1.3, and if the corresponding G2 peaks were distinctly separated, the SPF was calculated exclusively for the non-diploid stemline. In case of two or more non-diploid peaks, the SPF was calculated in the most prominent non-diploid stemline. SPF was not calculated if the corresponding G2 peak in the histogram could not be identified, or if the non-diploid stemline was small (G0/G1 < 15% of the total number of observations), or if the coefficient of variation (CV) of the G0/G1 peak exceeded 8%, or if the contribution of debris in the SPF compartment of the histogram was too extensive (Shankey et al, 1993). Nuclear aggregates were gated out using thresholding capabilities of the DNA analysis software in the Ortho 2140 data handling system.

***MultiCycle*** The SPF was calculated using the commercial MultiCycle software (version 3.1.1, Phoenix Flow System, San Diego, CA, USA). This software is based on the non-linear least square curve-fitting method described by Marquardt (1963). All G0/G1 peaks and G2 peaks in the DNA histogram were fitted by Gaussians and without restrictions on the CV values of the G2 peak (free from the CV of the G0/G1 peak). Furthermore, the S-phase compartment was always fitted by a zero order polynomial (y = constant). The SPF was calculated in five different ways:

1. Without any background correction (= subtraction) (in Tables 2 and 3 denoted 'no corr').
2. With a cell-related exponential background correction (in Tables 2 and 3 denoted 'exp').
3. With a cell-related exponential background correction including nuclear aggregate compensation based on the random probabilistic nuclear aggregation theory, as described in the MultiCycle operators manual (in Tables 2 and 3 denoted 'exp-clust').
4. With a sliced nuclei background fit including an exponential function for the left-most events in the DNA histogram (in Tables 2 and 3 denoted 'slice').
5. With a sliced nuclei background fit including an exponential function for the left-most events in the DNA histogram including nuclear aggregate compensation based on the random probabilistic nuclear aggregation theory, as described in the MultiCycle operators manual (in Tables 2 and 3 denoted 'slice-clust').

Criteria for S-phase calculation were used according to the guidelines in Baldetorp et al (1995) and the recommendations described in the MultiCycle operators manual.

### Statistics

The data were analysed using the $\chi^2$ test with the Yates continuity correction when indicated. Analyses of MFSR were univariately performed with Kaplan–Meier methods and the generalized Wilcoxon test.

### RESULTS

### Successful calculations

SPF could be calculated in 159 of the 259 patients using the Baisch method; the reasons for failure were high background distribution and/or a small non-diploid peak and/or a high CV. SPF could be calculated in 253 patients using the MultiCycle program, the reasons were a high CV and/or no G2 peak. The mean SPF values varied between 6.0% and 13.6% and the median values varied between 4.0% and 11.5% depending on which method was used. Regardless of method used, the median SPF values were at least three times as high for aneuploid tumours as for euploid tumours (Table 2).

### Prognostic separation

For each method the prognostic strength was analysed using the cut-off at (1) the median SPF value, (2) at 3.0%, and (3) at the optimum for each method. The cut-off value chosen was the one which gave the largest separation in MFSR at 5 years between the group with good prognosis and the one with poor prognosis. This cut-off value varied between 3.0% and 9.0%; lowest for Baisch and for MultiCycle with exponential background correction, and highest for MultiCycle without background correction and for MultiCycle with background correction for sliced nuclei.

The best separation in 5-year MFSR between the good prognosis group and the poor prognosis group was seen using the Baisch method with cut-off value at 3.0%; 0.94 vs 0.53 (Table 2).

*© Cancer Research Campaign 1999*

**Table 1** Clinico-pathologic data, metastasis-free survival, and crude metastasis rates in 259 surgically treated patients with soft tissue sarcoma of extremity and trunk wall

| Factor/Criteria | *n* | 5-year MFSR | Crude metastasis *n* |
|---|---|---|---|
| Age | | | |
| ≤ 64 years | 133 | 0.64 | 53 |
| > 64 years | 126 | 0.60 | 47 |
| Sex | | | |
| Male | 145 | 0.60 | 58 |
| Female | 114 | 0.66 | 42 |
| Localization | | | |
| Upper extremity proximal | 36 | 0.66 | 15 |
| Upper extremity distal | 17 | 0.73 | 4 |
| Trunk wall | 34 | 0.49 | 16 |
| Lower extremity proximal | 125 | 0.60 | 51 |
| Lower extremity distal | 47 | 0.74 | 14 |
| Depth | | | |
| Subcutaneous | 78 | 0.76 | 19 |
| Deep-seated | 181 | 0.57 | 81 |
| Tumour size (cm) | | | |
| 1–5 | 88 | 0.77 | 21 |
| 6–10 | 110 | 0.61 | 44 |
| 11–15 | 38 | 0.49 | 21 |
| 16 and larger | 23 | 0.35 | 14 |
| Histotype | | | |
| MFH | 96 | 0.65 | 33 |
| Leiomyosarcoma | 48 | 0.62 | 18 |
| Liposarcoma | 39 | 0.76 | 12 |
| Synovial sarcoma | 17 | 0.71 | 6 |
| Other than above | 59 | 0.48 | 31 |
| Malignancy grade | | | |
| I | 13 | 1.00 | 0 |
| II | 38 | 0.84 | 7 |
| III | 82 | 0.69 | 29 |
| IV | 126 | 0.47 | 64 |
| Tumour necrosis | | | |
| No | 129 | 0.82 | 24 |
| Yes | 127 | 0.41 | 76 |
| Vascular invasion | | | |
| No | 188 | 0.71 | 56 |
| Yes | 67 | 0.37 | 43 |

Microscopic tumour necrosis determined in 256 patients. Vascular invasion determined in 255 patients. MFSR = metastasis-free survival rate; MFH = malignant fibrous histocytoma.

### Late metastasis

The Baisch method was better than the others in identifying patients with late first metastasis; four out of seven patients with SPF 0–3.0% according to Baisch had their first metastases diagnosed after 5 years. No other method could demonstrate this phenomenon (Table 2).

### Subgroup analysis

In the 159 patients in whom SPF calculation according to Baisch was successful, the best prognostic separation in metastasis-free survival at 5 years was found using MultiCycle with cell-related exponential background correction and with nuclear aggregate compensation based on the random probabilistic nuclear aggregation theory (‘exp-clust’). The Baisch method again was the best for identification of patients with late first metastasis (Table 3).

### DISCUSSION

SPF has been identified as a prognostic factor in several malignancies: breast cancer, non-small cell lung cancer, colorectal cancer, carcinoma of the ovary, endometrial cancer, prostate cancer (Merkel and McGuire, 1990; Sigurdsson et al, 1990; Gudmundsson et al, 1995; Bratt et al, 1996) and recently in STS (Huuhtanen et al, 1996; Collin et al, 1997; Gustafson et al, 1997).

The quality of the DNA histogram may depend on several factors:

1. The contribution from cellular debris may cause different background patterns in the histogram depending on tumour type
2. The background may vary depending on whether the specimen is derived from a fresh–frozen or paraffin-embedded material.

For S-phase calculation specifically, the question arises how to handle the background contribution from cellular debris and/or nuclear aggregates. The traditional non-parametric model described by Baisch et al (1975) is based on finding a region of the S-phase compartment minimally influenced by debris and aggregates (Baldetorp et al, 1995). Thus, a visual correction for obvious

**Table 2** Flow cytometric SPF values and prognostic strength in 259 soft tissue sarcomas of the loco-motor system, calculated with different methods

| Method | A | B | C | D | E | F | G | H | I |
|---|---|---|---|---|---|---|---|---|---|
| Baisch | 159 | 7.8 | 6.0 | 0.1–25 | e 3.4<br>ae 11.0 | 3 | 46/113 | 0.94/0.53 | 4/7 |
| No corr | 253 | 13.6 | 11.5 | 0–47.5 | e 4.0<br>ae 16.4 | 9 | 102/151 | 0.76/0.53 | 6/30 |
| Exp | 253 | 9.5 | 7.1 | 0–45.1 | e 2.4<br>ae 10.8 | 3 | 77/176 | 0.82/0.53 | 4/17 |
| Exp-clust | 253 | 6.0 | 4.0 | 0–33.1 | e 1.5<br>ae 6.1 | 8 | 188/65 | 0.67/0.50 | 7/67 |
| Slice | 253 | 10.5 | 8.5 | 0–44.5 | e 2.8<br>ae 12.5 | 9 | 135/118 | 0.75/0.47 | 6/39 |
| Slice-clust | 253 | 6.5 | 4.5 | 0–33.9 | e 1.9<br>ae 6.8 | 5 | 135/118 | 0.72/0.51 | 5/41 |

A = total number possible to calculate; B = mean SPF value (%); C = median SPF value (%); D = range (%); E = median SPF value (%); F = optimal cut-off values (%) found after step-wise analysis of maximum separation between curves in a Kaplan–Meier plot; G = number of patients in groups; H = 5-year metastasis’ free survival rate with optimal cut-off, ‘good’ group/‘bad’ group; I = in ‘good’ group: number of patients with metastasis after 5 years/total number of patients with metastasis. No corr = no background correction. Exp = exponential background correction. Exp-clust = exponential background correction with cluster compensation. Slice = correction for slices. Slice-clust = correction for slices and clusters. e = euploid. ae = aneuploid. SPF = S-phase fraction.

© Cancer Research Campaign 1999

**Table 3** Flow cytometric SPF values and prognostic strength in 159 soft tissue sarcomas of the loco-motor system, calculated with different methods

| Method | Optimal cut-off % 'good'/'bad' group | 5-year MFSR rate with optimal cut-off 'good'/'bad' group | *P*-value for difference at 5 years | Metastasis after 5 years 'good' group |
|---|---|---|---|---|
| Baisch | 0–3/3.1– | 0.94/0.53 | < 0.0001 | 4/7 |
| No corr | 0–9/9.1– | 0.76/0.49 | < 0.0001 | 5/26 |
| Exp | 0–3/3.1– | 0.82/0.52 | < 0.0001 | 4/16 |
| Exp-clust | 0–8/8.1– | 0.72/0.28 | < 0.0001 | 5/40 |
| Slice | 0–9/9.1– | 0.74/0.41 | < 0.0001 | 5/34 |
| Slice-clust | 0–5/5.1– | 0.75/0.47 | < 0.0001 | 5/29 |

No corr = no background correction. Exp = exponential background correction. Exp-clust = exponential background correction with cluster compensation. Slice = correction for slices. Slice-clust = correction for slices and clusters. *n* = number of successful calculations. Optimal cut-off found after step-wise analysis of maximum separation between curves in a Kaplan–Meier plot. MFSR = metastasis-free survival rate. SPF = S-phase fraction.

background contribution is performed. This method has been reported to be of prognostic value in several malignancies other than STS: breast cancer, endometrial cancer and prostate cancer. However, this model requires considerable experience with non-automatic DNA analysis interpretation for objective and reproducible results (Baldetorp et al, 1995; Stål and Baldetorp, 1998). In the parametric models all compartments of the DNA histogram are mathematically estimated by e.g. a least square curve-fitting technique, which may produce objective and reproducible results on all parameters of the DNA histogram. However, regardless of the method used, it is impossible to validate the correctness in the background correction. Thus, the final SPF calculation may well be data without relevance to tumour kinetics and/or clinical course. Furthermore, it is difficult to define objective criteria regarding background distribution and/or the size of the DNA stemline to be analysed to permit a reliable calculation of SPF. Attempts to recommend different types of mathematical correction models for SPF in various settings have been made (Beck, 1980; Bagwell et al, 1991; Rabinovitch, 1991), and at a consensus conference experiences were pooled into general recommendations and guidelines for clinical DNA flow cytometry (Shankey et al, 1993).

Huuhtanen et al (1996) analysed 155 paraffin-embedded STS, using MultiCycle with the sliced nuclei option for background subtraction. SPF could be calculated in all patients, and three subgroups were used. A high SPF predicted a shorter survival in patients with diploid tumours. Gustafson et al (1997) analysed 260 paraffin-embedded tumours using the Baisch method. SPF could be calculated in 160 of these tumours, and two subgroups were used. High SPF (> 3.0%) was an independent prognostic factor for metastasis. Collin et al (1997) found SPF ≥ 4% calculated according to the Baisch method to be an independent prognostic factor for tumour death in 132 patients with STS. Also in this series the reported failure rate was 40%, despite the use of frozen material.

We have earlier shown that patients in whom calculation of SPF according to Baisch failed have a different distribution of histotypes and malignancy grades, and a worse 5-year MFSR, implying that they may belong to a different subset of STS patients (Gustafson et al, 1997). In the present series, using the MultiCycle software, it was possible to calculate the SPF in the majority of cases where the Baisch method failed. However, the survival for the 'poor prognosis' group using MultiCycle was not significantly separated from the 'poor prognosis' group using the Baisch method. On the other hand, the survival for the 'good prognosis' group was worse using MultiCycle than using Baisch. It is obvious that the Baisch method, using international consensus guidelines on when not to calculate SPF, inherently selects a group of patients with good prognosis. In a large series of breast cancers, SPF calculation using the Baisch model gave results with higher prognostic significance that models using background correction (Baldetorp et al, 1998).

The possibility to identify patients who will metastasize late is of clinical importance; they may be at risk of developing metastasis and/or local recurrence for a longer time-period, and may require a longer follow-up period (Gustafson et al, 1997).

We conclude that for clinical use in STS, despite a high failure rate, SPF should be calculated using the Baisch planimetric method when using paraffin archival material. This model gives the best prognostic separation in metastasis-free survival, and also the possibility to identify patients who require a longer follow-up.

### ACKNOWLEDGEMENTS

Grants were received from the Medical Faculty, Lund University, The Swedish Cancer Society, The Swedish Medical Research Council, The Berta Kamprad Foundation, The Gunnar, Arvid and Elisabeth Nilsson Foundation, The IngaBritt and Arne Lundberg Foundation, and The Lund University Hospital Foundation. All data are available on disks.

### REFERENCES

Bagwell CB, Mayo SW, Whetstone SD, Hitchcox SA, Baker DR, Herbert DJ, Weawer DL, Jones MA and Lovett EJ III (1991) DNA histogram debris theory and compensation. *Cytometry* **12**: 107–118

Baisch H, Gohde W and Linden WA (1975) Analysis of PCP-data to determine the fraction of cells in the various phases of cell cycle. *Radiat Environ Biophys* **12**: 31–39

Baldetorp B, Dalberg M, Holst U and Lindgren G (1989) Statistical evaluation of cell kinetic data from DNA flow cytometry (FCM) by the EM algorithm. *Cytometry* **10**: 695–705

Baldetorp B, Bendahl P-O, Fernö M, Alanen K, Delle U, Falkmer U, Hansson-Aggesjö B, Höckenström T, Lindgren A, Mossberg L, Nordling S, Sigurdsson H, Stål O and Visakorpi T (1995) Reproducibility in DNA flow cytometric analysis of breast cancer: comparison of 12 laboratories results for 67 sample homogenates. *Cytometry* **22**: 115–127

Baldetorp B, Stål O, Ahrens O, Cornelisse C, Corwer W, Falkmer U and Fernö M (1998) Different calculation methods for flow cytometric S-phase fraction: prognostic implications in breast cancer? *Cytometry* **33**: 385–393

Beck HP (1980) Evaluation of flow cytometric data of human tumours. Correction procedures for background and cell aggregates. *Cell Tissue Kinet* **13**: 173–181

 *© Cancer Research Campaign 1999*

Bratt O, Andersson H, Bak-Jensen E, Baldetorp B and Lundgren R (1996) Metaphase cytogenetics and DNA flow cytometry with analysis of S-phase fraction in prostate cancer: influence on prognosis. *Urology* **47**: 218–224

Collin F, Chassevent A, Bonichon F, Bertrand G, Terrier P and Coindre J-M (1997) Flow cytometric DNA content analysis of 185 soft tissue neoplasms indicates that S-phase fraction is a prognostic factor for sarcomas. *Cancer* **79**: 2371–2379

Gudmundsson TE, Högberg T, Alm P, Anderson H, Baldetorp B, Fernö M, Långström E and Killander D (1995) The prognostic information of DNA ploidy and S-phase fraction may vary with histologic grade in endometrial carcinoma. *Acta Oncologica* **34**: 803–812

Gustafson P (1994) Soft tissue sarcoma. Epidemiology and prognosis in 508 patients. *Acta Orthop Scand* **65** (suppl 259): 1–31

Gustafson P, Fernö M, Åkerman M, Baldetorp B, Willén H, Killander D and Rydholm A (1997) Flow cytometric S-phase fraction in soft-tissue sarcoma: prognostic importance analysed in 160 patients. *Br J Cancer* **75**: 94–100

Huuhtanen R, Blomqvist C, Wiklund T, Virolainen M, Elomaa I, Pan Y and Tribukait B (1996) S-phase fraction of 155 soft tissue sarcomas. Correlation with clinical outcome. *Cancer* **77**: 1815–1822

Marquardt DW (1963) An algoritm for least-squares estimation of nonlinear parameters. *Soc Ind Appl Math* **11**: 431–441

Merkel DE and McGuire WL (1990) Ploidy, proliferative activity and prognosis. DNA flow cytometry of solid tumours. *Cancer* **65**: 1194–1205

Rabinovitch PS (1991) Numerical compensation for the effects of cell clumping on DNA content histograms. *Cytometry* **4**(suppl): 27

Schutte B, Reynders MMJ, Bosman FT and Blijham GH (1985) Flow cytometric determination of DNA ploidy level in nuclei isolated from paraffin-embedded tissue. *Cytometry* **6**: 26–30

Shankey TV, Rabinovitch PS, Bagwell B, Bauer KD, Duque RE, Hedley DW, Mayall BH and Wheeless L (1993) Guidelines for implementation of clinical DNA cytometry. *Cytometry* **14**: 472–477

Sigurdsson H, Baldetorp B, Borg Å, Dalberg M, Fernö M, Killander D and Olsson H (1990) Indicators of prognosis in node-negative breast cancer. *N Engl J Med* **322**: 1045–1053

Stål O and Baldetorp B (1998) S-phase fraction assessed by a variant of the rectangular model adapted to the flowcytometric DNA histogram profile. *Cytometry* **33**: 487–491

*© Cancer Research Campaign 1999*
